# Supplementary material for: A Systematic Review and Meta-Analysis on the Impact of Statin Treatment in HIV Patients on Antiretroviral Therapy
Source: Int J Environ Res Public Health. 2023 Apr 27;20(9):5668. doi: 10.3390/ijerph20095668 (PMC10177940; doi:10.3390/ijerph20095668)
Supplement: Supplementary file 1 [file ijerph-20-05668-s001.zip › ijerph-2305691-supplementary.pdf]

Systematic Review

A systematic Review and Meta-Analysis on the Impact of Statin Treatment in HIV Patients on Antiretroviral Therapy

Kabelo Mokgalaboni [1,*], Wendy Nokhwezi Phoswa [1], Samantha Yates [1], Sogolo Lucky Lebelo [1], Sphiwe Madiba [2] and Perpetua Modjadji [3]

1 Department of Life and Consumer Sciences, College of Agriculture and Environmental Sciences, University of South Africa, Florida Campus, Johannesburg 1709, South Africa; phoswwn@unisa.ac.za (W.N.P.); blakes@unisa.ac.za (S.Y.); lebelol@unisa.ac.za (S.L.L.)
2 Faculty of Health Sciences, University of Limpopo, Polokwane 0700, South Africa; sphiwe.madiba@ul.ac.za
3 Non-Communicable Disease Research Unit, South African Medical Research Council, Cape Town 7505, South Africa; perpetua.modjadji@mrc.ac.za
* Correspondence: mokgak@unisa.ac.za

Search strategy

Table S1A: Search strategy adapted on PubMed on the 18th February, 2023

**MesH Terms**	**Number of records**
Statins	34117
Atorvastatin	7321
Rosuvastatin	2,884
Pitavastatin	0
Lovastatin	11888
HIV	106721
**Final search with restrictions**<br>**Randomised controlled trials**	
((((((Statins[MeSH Terms]) OR (Atorvastatin[MeSH Terms])) OR (Rosuvastatin[MeSH Terms])) OR (Pravastatin[MeSH Terms])) OR (Pitavastatin[MeSH Terms])) OR (Lovastatin[MeSH Terms])) AND (HIV[MeSH Terms]) Filters: Randomized Controlled Trial	15

Table S1B: Search strategy adapted on Scopus

**Search terms and strategy**	**Number of records**
Statin OR hmg-coa AND reductase AND inhibitors OR atorvastatin OR	710

fluvastatin OR simvastatin OR rosuvastatin OR lovastatin OR pitavastatin<br><br>AND hiv OR aids AND anti-retroviral AND therapy OR art AND (LIMIT-TO (LANGUAGE , "English")) AND (LIMIT TO (SRCTYPE , "j")) AND (LIMIT-TO (DOCTYPE , "ar"))	

Table S1C: Search strategy adapted on Cochrane Library

**Search terms and strategy**	**Number of records**
**Statins in HIV patients on Antiretroviral therapy in Title Abstract Keyword**	**70**

Table S2: Quality assessment according to a modified JADAD score guideline.

**Study**	**Was the study described as randomised?**	**Was double-blinded correct?**	**Loses and exclusions were described**	**The randomisation was adequate.**	**Total**	**Judgement**
Bonnet[1]	1	1	1	0	3	Good
Hearps[2]	1	1	0	0	2	Fair
Hileman[3]	1	1	1	1	4	Excellent
Hileman [4]	1	0	1	1	3	Good
Hu¨rlimann[5]	1	1	0	0	2	Fair
Kamari[6]	1	1	1	1	4	Excellent
Longenecker[7]	1	1	1	1	4	Excellent
Mallon[8]	1	1	0	1	3	Good
Morrison[9]	1	1	0	1	3	Good
Nakanjako[10]	1	1	1	1	4	Excellent
Nixon[11]	1	1	1	0	3	Good
Nou[12]	1	1	1	1	4	Excellent
Stein[13]	1	1	0	1	3	Good
Trevillyan[14]	1	1	1	1	4	Excellent
Wongprikorn[15]	1	1	0	1	3	Good
Eckard[16]	1	1	1	1	4	Excellent
Erlandson[17]	1	1	1	0	3	Good
Funderburg[18]	1	1	1	1	4	Excellent
Do [19]	1	0	1	1	3	Good
defilippi[21]	1	1	1	0	3	Good
**total**	**20**	**18**	**14**	**14**	**66**	

Study or Subgroup	Statin Mean	Statin SD	Statin Total	Placebo Mean	Placebo SD	Placebo Total	Weight	Std. Mean Difference IV, Random, 95% CI	Year
Stein et al., 2004	537	55	20	537	55	20	4.7%	0.00 [-0.62, 0.62]	2004
Hu¨rlimann et al., 2006	464	74	29	472	81.5	29	5.2%	-0.10 [-0.62, 0.41]	2006
Mallon et al., 2006	502	245.19	16	442	138.15	17	4.4%	0.30 [-0.39, 0.98]	2006
Bonnet et al., 2007	446.25	56.28	12	512.5	64.68	9	3.4%	-1.06 [-2.00, -0.13]	2007
Funderburg et al., 2013	607.5	68	72	627	75.83	75	6.0%	-0.27 [-0.59, 0.06]	2013
Do et al., 2014	595	91.5	64	627	108	59	5.9%	-0.32 [-0.67, 0.04]	2014
Eckard et al., 2014	608	68	72	627	75.83	75	6.0%	-0.26 [-0.59, 0.06]	2014
Nakanjako et al., 2015	2.03	0.5	15	2.8	0.76	15	4.0%	-1.16 [-1.95, -0.38]	2015
Erlandson et al., 2015	6.1	0.68	72	6.3	0.76	75	6.0%	-0.28 [-0.60, 0.05]	2015
Hileman et al., 2015	644	287	72	636	314	75	6.0%	0.03 [-0.30, 0.35]	2015
Morrison et al., 2016	608	84.67	72	627	75.83	75	6.0%	-0.24 [-0.56, 0.09]	2016
Nou et al., 2016	522	263	19	590	289	21	4.7%	-0.24 [-0.86, 0.38]	2016
Wongprikorn et al., 2016	641.9	196.5	12	718.1	181.2	12	3.9%	-0.39 [-1.20, 0.42]	2016
Longenecker et al., 2016	608	68	72	627	75.83	75	6.0%	-0.26 [-0.59, 0.06]	2016
Nixon et al., 2017	587	89.75	46	545	66.25	48	5.7%	0.53 [0.12, 0.94]	2017
Hileman et al., 2017	643.57	286.95	72	635.73	314.22	75	6.0%	0.03 [-0.30, 0.35]	2017
Kamari et al., 2019	644	287	72	636	314	75	6.0%	0.03 [-0.30, 0.35]	2019
Trevillyan et al., 2021	693	259	44	550	254	40	5.5%	0.55 [0.12, 0.99]	2021
Hearps et al., 2021	746	82.25	33	505	87.5	36	4.4%	2.80 [2.13, 3.48]	2021
**Total (95% CI)**			**886**			**906**	**100.0%**	**-0.01 [-0.25, 0.23]**	

Heterogeneity: Tau² = 0.23; Chi² = 108.94, df = 18 (P < 0.00001); I² = 83%
Test for overall effect: Z = 0.07 (P = 0.95)

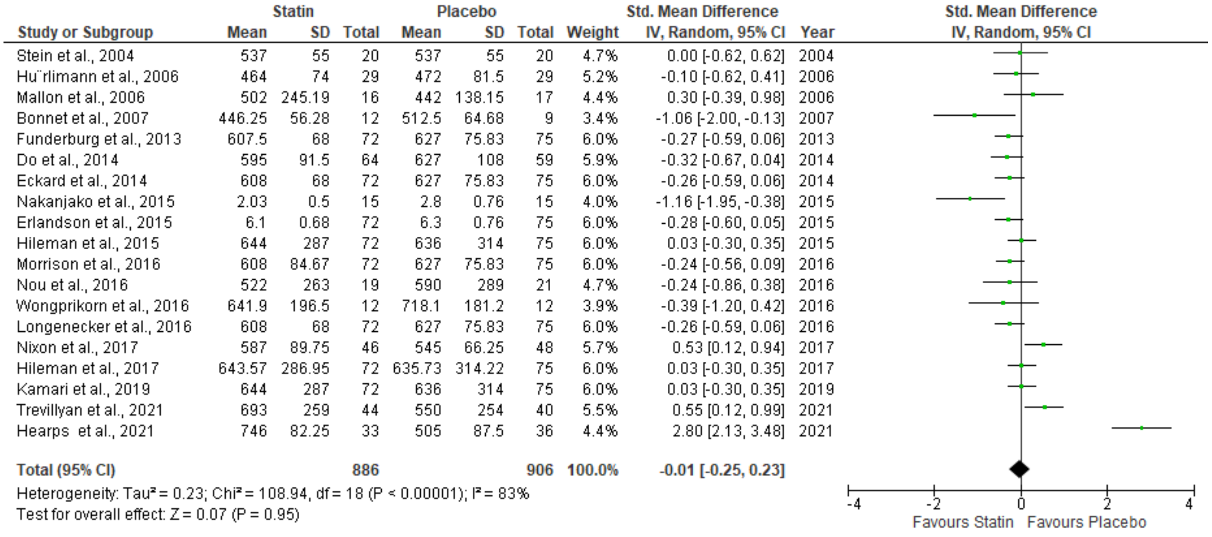

Figure S1: Effect of statin on baseline CD4 count in PLHIV on ART. I² statistic was used to assess statistical heterogeneity. The probability value of less than 0.05 was considered statistically significant.

Study or Subgroup	Statin Events	Statin Total	Placebo Events	Placebo Total	Weight	Risk Ratio M-H, Random, 95% CI	Year
Stein et al., 2004	15	15	15	15	5.3%	1.00 [0.88, 1.13]	2004
Funderburg et al., 2013	56	72	58	75	2.7%	1.01 [0.85, 1.20]	2013
Do et al., 2014	52	64	49	59	3.0%	0.98 [0.83, 1.15]	2014
Eckard et al., 2014	56	72	58	75	2.7%	1.01 [0.85, 1.20]	2014
Erlandson et al., 2015	56	72	58	75	2.7%	1.01 [0.85, 1.20]	2015
Hileman et al., 2015	56	72	58	75	2.7%	1.01 [0.85, 1.20]	2015
Longenecker et al., 2016	56	72	58	75	2.7%	1.01 [0.85, 1.20]	2016
Morrison et al., 2016	56	72	58	75	2.7%	1.01 [0.85, 1.20]	2016
Nou et al., 2016	16	19	17	21	1.0%	1.04 [0.78, 1.38]	2016
Wongprikorn et al., 2016	12	12	11	12	1.6%	1.09 [0.87, 1.36]	2016
Hileman et al., 2017	56	72	58	75	2.7%	1.01 [0.85, 1.20]	2017
Nixon et al., 2017	46	46	47	48	24.6%	1.02 [0.96, 1.08]	2017
deFilippi et al., 2018	16	19	16	21	0.9%	1.11 [0.81, 1.50]	2018
Kamari et al., 2019	55	72	57	75	2.5%	1.01 [0.84, 1.20]	2019
Hearps et al., 2021	27	31	33	36	2.9%	0.95 [0.80, 1.12]	2021
Trevillyan et al., 2021	44	44	40	40	39.0%	1.00 [0.96, 1.05]	2021
**Total (95% CI)**		**826**		**852**	**100.0%**	**1.01 [0.98, 1.04]**	
Total events	675		691				

Heterogeneity: Tau² = 0.00; Chi² = 1.74, df = 15 (P = 1.00); I² = 0%
Test for overall effect: Z = 0.46 (P = 0.65)

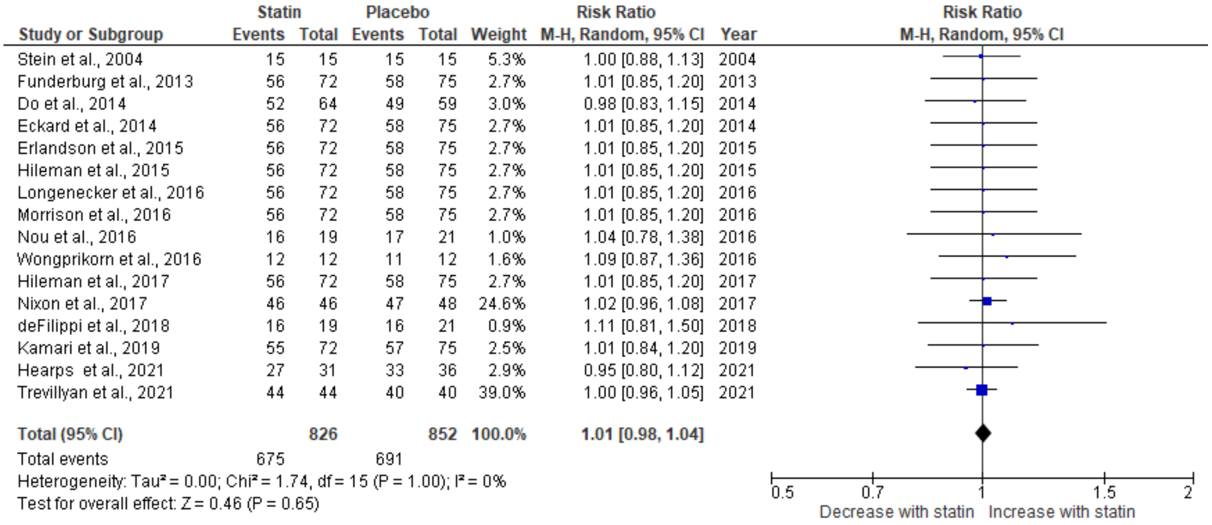

Figure S2: Effect of statin on undetectable viral load. I² statistic was used to assess statistical heterogeneity. The probability value of less than 0.05 was considered statistically significant.

Study or Subgroup	Statin Mean	Statin SD	Statin Total	Placebo Mean	Placebo SD	Placebo Total	Weight	Std. Mean Difference IV, Random, 95% CI
**1.1.1 Atorvastatin**								
deFilippi et al., 2018	522	263	19	590	289	21	4.5%	-0.24 [-0.86, 0.38]
Nixon et al., 2017	587	89.75	46	546	66.25	48	5.4%	0.52 [0.11, 0.93]
Nou et al., 2016	522	263	19	590	289	21	4.5%	-0.24 [-0.86, 0.38]
**Subtotal (95% CI)**			**84**			**90**	**14.4%**	**0.06 [-0.50, 0.61]**
Heterogeneity: Tau² = 0.16; Chi² = 6.07, df = 2 (P = 0.05); I² = 67%								
Test for overall effect: Z = 0.20 (P = 0.84)								
**1.1.2 Rosuvastatin**								
Do et al., 2014	595	91.5	64	627	108	55	5.6%	-0.32 [-0.68, 0.04]
Eckard et al., 2014	606	68	72	627	75.83	75	5.8%	-0.29 [-0.61, 0.04]
Erlandson et al., 2015	6.1	0.68	72	6.3	0.75	75	5.8%	-0.28 [-0.60, 0.05]
Funderburg et al., 2013	607.5	68	72	627	75.83	75	5.8%	-0.27 [-0.59, 0.06]
Hearps et al., 2021	746	82.25	33	505	87.5	36	4.3%	2.80 [2.13, 3.48]
Hileman et al., 2015	644	287	72	636	314	75	5.8%	0.03 [-0.30, 0.35]
Hileman et al., 2017	643.57	286.99	72	635.73	314.22	75	5.8%	0.03 [-0.30, 0.35]
Kamari et al., 2019	464	74	29	472	81.5	29	5.0%	-0.10 [-0.62, 0.41]
Longenecker et al., 2016	644	287	72	636	314	75	5.8%	0.03 [-0.30, 0.35]
Morrison et al., 2016	608	84.67	72	627	75.83	75	5.8%	-0.24 [-0.56, 0.09]
Nakanjako et al., 2015	2.03	0.5	15	2.8	0.76	15	3.8%	-1.16 [-1.95, -0.38]
Trevillyan et al., 2021	693	259	44	550	254	40	5.3%	0.55 [0.12, 0.99]
**Subtotal (95% CI)**			**689**			**700**	**64.6%**	**0.04 [-0.28, 0.36]**
Heterogeneity: Tau² = 0.27; Chi² = 93.55, df = 11 (P < 0.00001); I² = 88%								
Test for overall effect: Z = 0.25 (P = 0.80)								
**1.1.3 Pravastatin**								
Bonnet et al., 2007	446.25	56.28	12	512.5	64.68	9	3.2%	-1.06 [-2.00, -0.13]
Hu¨rlimann et al., 2006	464	74	29	472	81.5	29	5.0%	-0.10 [-0.62, 0.41]
Mallon et al., 2006	502	245.19	16	442	138.15	29	4.5%	0.32 [-0.29, 0.94]
Stein et al., 2004	537	245.85	20	537	245.85	20	4.5%	0.00 [-0.62, 0.62]
**Subtotal (95% CI)**			**77**			**87**	**17.3%**	**-0.12 [-0.57, 0.34]**
Heterogeneity: Tau² = 0.11; Chi² = 5.95, df = 3 (P = 0.11); I² = 50%								
Test for overall effect: Z = 0.51 (P = 0.61)								
**1.1.4 Pitavastatin**								
Wongprikorn et al., 2016	641.9	196.5	12	718.1	181.2	12	3.7%	-0.39 [-1.20, 0.42]
**Subtotal (95% CI)**			**12**			**12**	**3.7%**	**-0.39 [-1.20, 0.42]**
Heterogeneity: Not applicable								
Test for overall effect: Z = 0.94 (P = 0.35)								
**Total (95% CI)**			**862**			**889**	**100.0%**	**-0.01 [-0.25, 0.23]**
Heterogeneity: Tau² = 0.23; Chi² = 108.21, df = 19 (P < 0.00001); I² = 82%								
Test for overall effect: Z = 0.07 (P = 0.94)								
Test for subgroup differences: Chi² = 1.18, df = 3 (P = 0.76), I² = 0%								

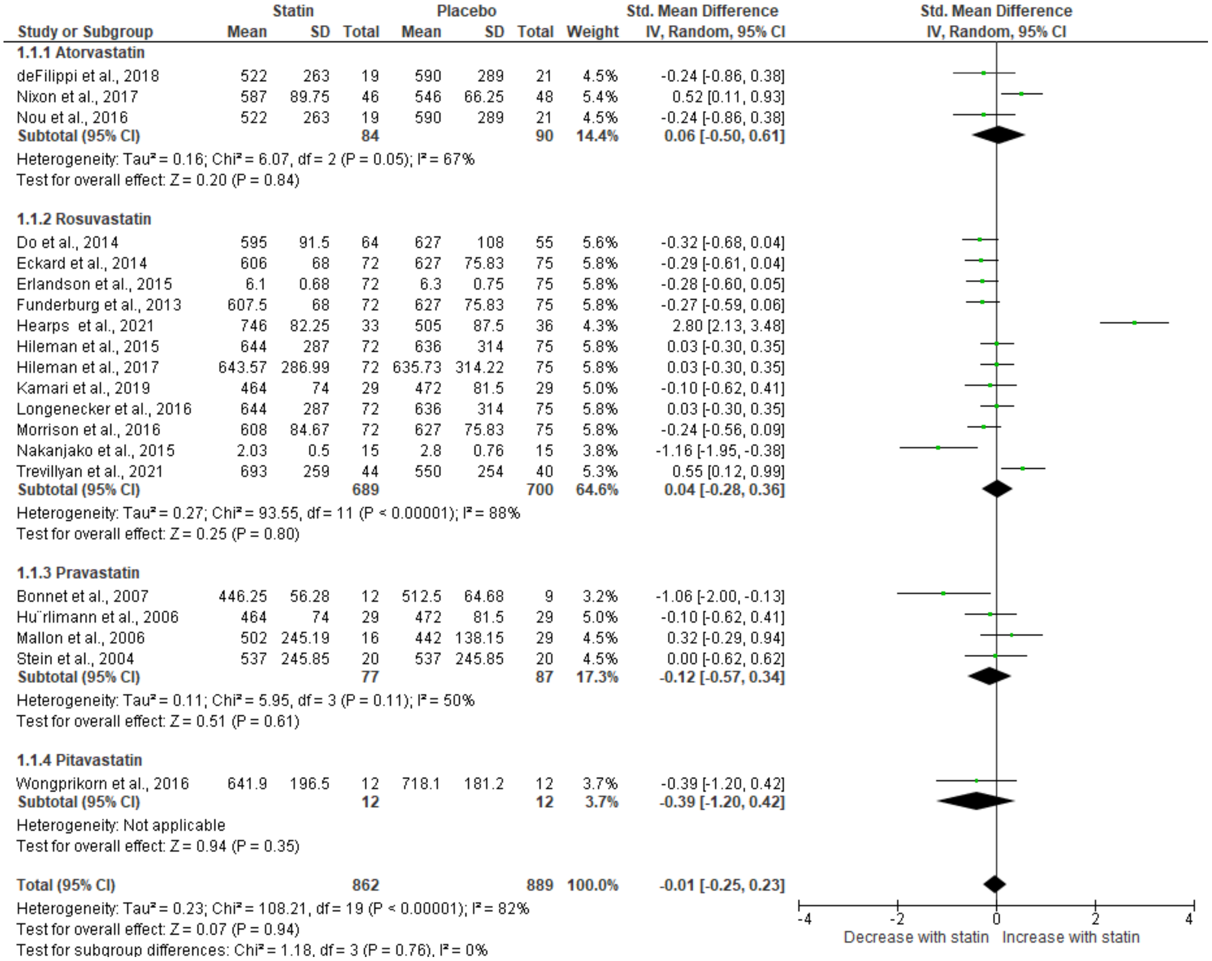

Figure S3: Subgroup analysis for CD4 count baseline according to statins. I² statistic was used to assess statistical heterogeneity. The probability value of less than 0.05 was considered statistically significant.

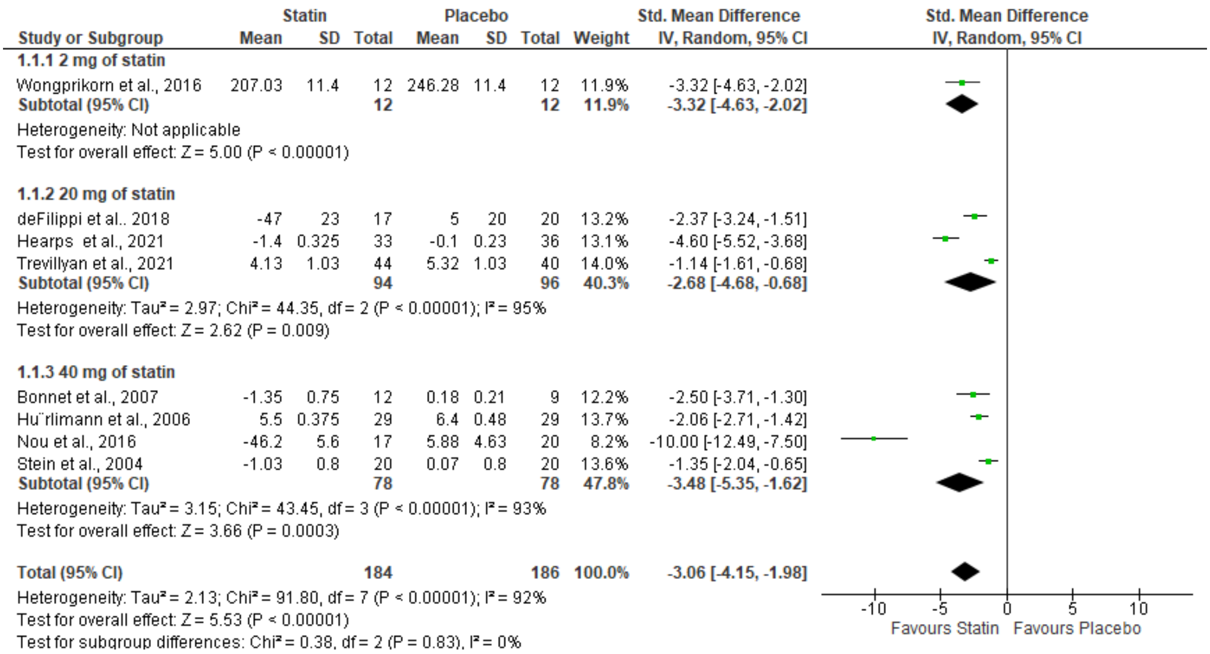

Figure S4: Subgroup total cholesterol according to doses of statin. I^2 statistic was used to assess statistical heterogeneity. The probability value of less than 0.05 was considered statistically significant.

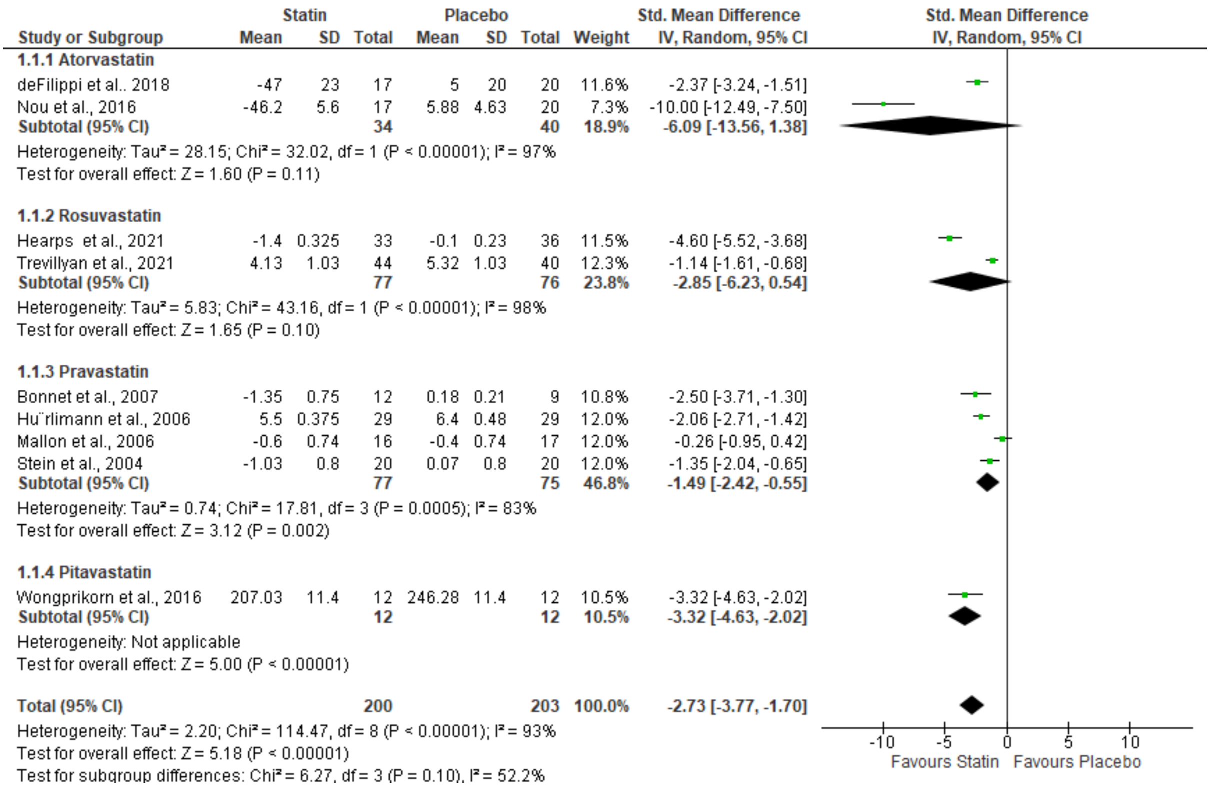

Figure S5: Subgroup total cholesterol according to the form of statin. I^2 statistic was used to assess statistical heterogeneity. The probability value of less than 0.05 was considered statistically significant.

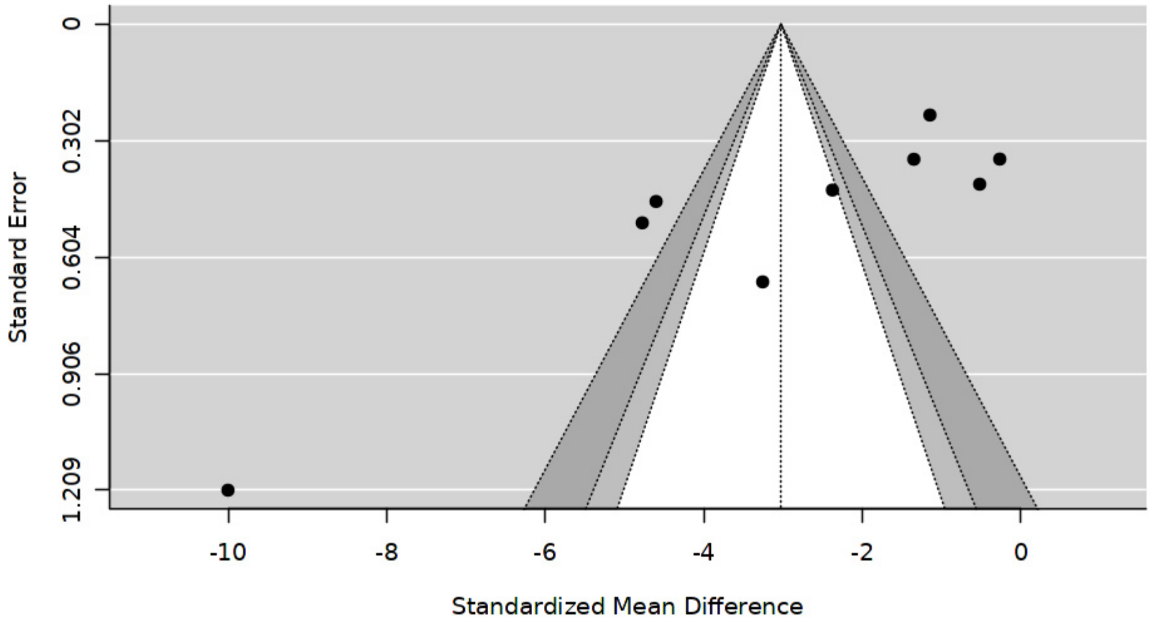

Figure S6: Publication bias for total cholesterol using a funnel plot.
